# Effects of Moderate Enzymatic Hydrolysis on Structure and Functional Properties of Pea Protein

**DOI:** 10.3390/foods11152368

**Published:** 2022-08-07

**Authors:** Xixiang Shuai, Lizhi Gao, Qin Geng, Ti Li, Xuemei He, Jun Chen, Chengmei Liu, Taotao Dai

**Affiliations:** 1 State Key Laboratory of Food Science and Technology, Nanchang University, Nanchang 330047, China; 2 Guangxi Academy of Agricultural Sciences, Nanning 530007, China

**Keywords:** pea protein, moderate enzymatic hydrolysis, structure properties, functional properties

## Abstract

Pea protein (PP) was moderately hydrolyzed using four proteolytic enzymes including flavourzyme, neutrase, alcalase, and trypsin to investigate the influence of the degree of hydrolysis (*DH*) with 2%, 4%, 6%, and 8% on the structural and functional properties of PP. Enzymatic modification treatment distinctly boosted the solubility of PP. The solubility of PP treated by trypsin was increased from 10.23% to 58.14% at the 8% *DH*. The results of SDS-PAGE indicated the protease broke disulfide bonds, degraded protein into small molecular peptides, and transformed insoluble protein into soluble fractions with the increased *DH*. After enzymatic treatment, a bathochromic shift and increased intrinsic fluorescence were observed for PP. Furthermore, the total sulfhydryl group contents and surface hydrophobicity were reduced, suggesting that the unfolding of PP occurred. Meanwhile, the foaming and emulsification of PP were improved after enzymatic treatment, and the most remarkable effect was observed under 6% *DH*. Moreover, under the same *DH*, the influence on the structure and functional properties of PP from large to small are trypsin, alcalase, neutrase and flavourzyme. This result will facilitate the formulation and production of natural plant-protein-based products using PP.

## 1. Introduction

In recent years, the use of plant proteins as an alternative to animal proteins has attracted considerable consumer interest due to their wide availability, affordability, hypoallergenicity, cholesterol-lowering effects, and ease of digestion [1]. Pea protein (PP) is a valuable plant protein with the advantages of being hypoallergenic, non-GMO, amino-acid-balanced and rich in lysine [2,3]. The amino acid ratio of PP is balanced, the content of the other seven kinds of human essential amino acids except methionine is close to the recommended model value of FAO and WHO, and it is easy to digest and absorb. Meanwhile, the lysine content is higher than other plant proteins.

Although PP possesses high nutritional value, its application in formula food is limited due to its poor water solubility and limited functional properties [3,4]. In order to overcome these drawbacks, physical modification, chemical modification, and enzymatic modification have been applied to improve the properties of PP. Physical modification, including heating, magnetic field, freezing, ultrasound, and microfluidization, has been reported to improve the structural, functional, and nutritional value of proteins [5,6]. Although physical modification does not involve the addition of exogenous substances, it is not widely used because the modification effect is limited [7]. Chemical modification was considered as an effective method to improve the functional properties of protein by introducing new organic groups into the protein molecule to break or polymerize the amino, carboxyl, sulfhydryl, or carbonyl groups on the main or side chains of the protein, resulting in changes in the structural and physicochemical properties of the protein [8]. However, chemical modification was also not widely used in the food industry because of the addition of chemicals that may be harmful to human health, such as toxicity.

Enzymatic hydrolysis is a promising technology in the protein field. Compared to physical and chemical modification, enzymatic modification is widely used because of the advantages of low by-products, high specificity, and easy control. It can convert native protein into peptides of various sizes and free amino acids [9]. Enzymatic hydrolysis was considered as one of the most promising methods for the modification of tailor-made protein preparations [10,11]. Arteaga et al. [12] used 11 enzymes to hydrolyze PP under different enzymolysis time, altering the molecular weight distribution and, thus, improving the technical functionality and sensory properties of PP. Klost et al. [13] investigated the ability of trypsin treatment to improve the poor solubility and interfacial properties of PP, and the potential to improve the overall stability of PP emulsions. Tamm et al. [14] found that trypsin treatment improved various properties of PP emulsions through characterization on molecular weight distribution, interfacial activity, expansive characterization, and emulsion properties. Klost et al. [15] discovered that the treatment of PP with trypsin improved the gel properties of fermented PP, which helped to develop PP yogurt alternatives for tailoring such gels. However, most studies mainly focused on single enzyme treatment and comparison of different enzyme treatments at the same enzymatic hydrolysis time. The effects of different enzyme treatments on PP at the same degree of enzymolysis (*DH*) were not clear. In addition, mild enzymatic hydrolysis modification does not improve the protein properties significantly, and excessive enzymatic hydrolysis modification would produce strong bitterness, thus affecting the organoleptic properties of PP [10,12]. Therefore, moderate enzymatic hydrolysis is required to improve the protein properties and produce less bitterness at the same time. Furthermore, systematic studies and comparative information on the effects of different *DH* on PP are limited.

Based on the above analysis, the present study focused on investigating the effects of different enzymes (flavourzyme, neutrase, alcalase, and trypsin) on the structural and functional properties of PP at the same *DH* under moderate enzymatic hydrolysis conditions.

## 2. Materials and Methods

### 2.1. Material

Pea protein (PP, purity ≥ 80%) was provided by the Shuangta Food Co., Ltd. (Qingdao, China). Enzymes including Alcalase 2.4 L FG (2.4 AU/Ml, endo protease), Neutrase 0.8 L (0.8 AU/g, endo protease), Trypsin (4000 U/g, acts on carboxyl side cleavage of lysine and arginine residues in polypeptide chains), and Flavourzyme 1000 L (1000 LAPU/g, mixture of endopeptidase and exopeptidase) were purchased from Novozymes (Bagsvaerd, Denmark). The SDS-PAGE kit and Micro Total Mercapto Assay Kit were purchased from Solarbio Science and Technology (Beijing, China). 1-anilino-8-naphthalene-sulfonate (ANS), sodium dodecyl sulfate, and ammonium peroxodisulfate were purchased from Sigma company (St. Louis, MO, USA). All other chemicals used were of analytical grade. Double-distilled water was used throughout the research.

### 2.2. Enzymatic Hydrolysis of PP

The hydrolysates of PP with different enzymatic hydrolysis degrees (*DH*) were prepared based on the study by Phoon et al. with some modification [16]. The specific steps were as follows: 10 g PP was dispersed in distilled water according to the ratio of protein to distilled water 1:15 (*w*/*w*), and stirred overnight at room temperature to make the protein fully hydrated. The pH of the aqueous solution of protein was adjusted to the optimum pH of the enzyme (alcalase and trypsin were pH 8; neutrase and flavourzyme were pH 7), and kept in a water bath (50 °C) for 15 min. Then, 1% enzyme (the ratio of enzyme to substrate was 1:100) was added for enzymolysis. In the process of enzymatic hydrolysis, 1 mol/L NaOH solution was added continuously to maintain the pH. After the enzymatic hydrolysis, the enzyme suspension was quickly moved into a water bath at 95 °C and heated for 15 min to inactivate the used enzyme. After cooling to ambient temperature, the pH adjusted to neutral.

### 2.3. Structure Properties

#### 2.3.1. Degree of Enzymolysis (*DH*)

The degree of enzymolysis (*DH*) is defined as the number of hydrolyzed peptide bonds relative to the number of peptide bonds per unit weight expressed as a percentage. The *DH* of PP was calculated using the pH-stat method [17]. In the enzymatic hydrolysis process, the *DH* of PP was calculated in real time by representing the volume of NaOH consumed by the sample. When the *DH* reached 2, 4, 6, and 8%, the enzymatic hydrolysis process was stopped and samples for subsequent experiments were obtained, Equation (1) is as follows:

(1) $$DH=\frac{{B\times N}_{b}}{{\alpha \times h}_{hot}{\times M}_{p}}\times 100\%$$

where *B* represents the volume of NaOH consumed by the sample; *N_b_* refers to the concentration of the calibrated NaOH; *α* is the degree of amino dissociation, which has different values under different enzymolysis conditions; *Mp* refers to the net protein content of PP; and *h_hot_* is the number of millimoles of peptide bonds per gram of protein, which is usually an empirical value. The *h_hot_* of PP is 7.55 mep/g.

#### 2.3.2. Intrinsic Fluorescence Spectroscopy

The intrinsic fluorescence spectra of PP and PP with different *DH* samples were determined according to previous papers by using an fluorescence spectrophotometer (F-7000, Hitachi, Tokyo, Japan) equipped with a 10 mm square quartz cell [18]. The emission spectra of PP samples were collected to investigate the effect of hydrolysis on the intrinsic fluorescence of PP. The critical parameters used in fluorescence were: excitation wavelength 295 nm, excitation slit 2.5 nm, emission slit 2.5 nm, and scan rate 1200 nm/min. Then, the emission spectra of PP samples were collected to investigate the effect of the enzyme on the intrinsic fluorescence of PP.

#### 2.3.3. Surface Hydrophobicity Measurements

The surface hydrophobicity of PP and PP with different *DH* samples was determined according to Li et al. [19]. ANS was used as a fluorescence probe; the excitation wavelength was set at 380 nm and emission spectra were collected from 390 to 660 nm. Both excitation and emission slits widths were fixed at 5 nm. Briefly, 20 μL 8 mM ANS was added into 3 mL samples with different protein concentrations (0.004%, 0.008%, 0.012%, 0.016%, and 0.020%). Under conditions of fluorescent probes present in excess, protein relative fluorescence intensity *F* was plotted against protein concentration *C*, and the slope of the line was represented as Equation (2):

(2) $$S_{0}=\frac{\Delta F}{\Delta C}$$

#### 2.3.4. Circular Dichroism (CD) Spectroscopy Studies

The secondary structure of PP and PP with different *DH* samples were measured utilizing a MOS-450 CD spectrometer (Claix, France) as in a previous study described by Li et al. [20]. The spectra of samples were collected from 180 to 260 nm. The PBS was measured as a background to correct the CD spectra. The secondary structure contents including α-helix, β-sheet, β-turn, and random coil of PP samples were calculated by the analytical approach of previous research using online CONTIN program [19].

#### 2.3.5. Morphology Observation

The microstructure of PP and PP with different *DH* samples was imaged utilizing a scanning electron microscope (Quanta-200, FEI Company, Eindhoven, The Netherlands). The freeze-dried protein powders were stuck onto one side of double adhesive tape attached to a circular specimen stub and then sputter-coated with a thin film of gold. Then, the microstructural images of the samples were captured under an accelerating voltage of 5.0 kV with magnification at 300-fold.

#### 2.3.6. Sodium Dodecyl Sulfate Polyacrylamide Gel Electrophoresis (SDS-PAGE)

SDS-PAGE under nonreducing conditions was performed to determine the protein fractions patterns of PP and PP with different *DH* samples according to He et al. [5]. Briefly, samples were dispersed in 10 mM phosphate buffer (pH 7) to obtain a 1 mg/mL protein sample. A 60 μL protein solution was mixed with 20 μL 4× loading buffer, followed by heating at 95 °C for 8 min. An aliquot containing 10 μg protein and the Thermo Scientific Page Ruler Prestained Protein Ladder (ranging from 11–245 kDa) were loaded to the specific cell. Electrophoresis was run for 15 min at 80 V for stacking gel (5%) and 55 min at 120 V for separating gel (12%). After electrophoresis, the gel was stained using 0.25% Coomassie Brilliant Blue in 50% methanol and 10% acetic acid for at least 50 min. Then, the destaining processing was performed using a water solution of 5% methanol and 7.5% acetic acid.

#### 2.3.7. Determination of Sulfhydryl Groups (SH)

The total sulfhydryl content of PP and PP with different *DH* samples was detected using a micro total mercapto assay kit (Sigma-Aldrich Co., Ltd., Shanghai, China) according to the method reported by Yang et al. [21]. Briefly, the sample of 0.1 g was weighed, and 1 mL of extract was added to prepare 10% homogenate. The sample was centrifuged at room temperature for 10 min at 8000× *g*, and the supernatant was taken to be measured. A certain amount of the samples and reagents was added according to the requirements of the kit, and the absorbance value was measured at a 412 nm.

#### 2.3.8. Amino Acid Composition Analysis

Amino acid composition was analyzed according to Xie et al. [22]. Briefly, 1.0 g samples, 10 mL hydrochloric acid (6 mol/L), and 1.0 g phenol were added to a sealed tube and the solution was treated with nitrogen for 15 min. The above samples were hydrolyzed for 24 h at 110 °C. The hydrolyzed samples were transferred to a 50 mL volumetric flask for constant volume and were then filtrated. The filtrated samples (1.0 mL) were evaporated to dryness at 60 °C in a water bath. Subsequently, the dried samples were diluted by sample diluent (3–5 mL, pH 2.2, 0.02 mol/L Sodium citrate buffer solution). The mixed samples were filtrated by a 0.22 μm filter membrane and then analyzed utilizing an automatic amino acid analyzer (S-433D, Sykam, Munich, German). The amino acid compositions for PP with different *DH* were presented as g/100 g protein.

#### 2.3.9. Hydrophobicity Analysis Based on Amino Acid Composition

The hydrophobicity of PP with different *DH* samples was determined according to previous research [17], Equations (3) and (4) are as follows:

(3) Q=∑ΔQi

(4) ΔQ=[(AAi/Mi)/(∑AAi/Mi)] ×Δf

where *AAi* refers to the amount of various amino acids in 100 mL of protein; *Mi* represents the molar mass of various amino acids; ∑*AAi/Mi* is the total number of moles of amino acids; Δ*f* refers to the free energy values of amino acid side chains; and Δ*Q* is the free energy for the transfer of an amino acid side chain from ethanol to water.

#### 2.3.10. Sensory Evaluation

The color, taste, beany flavor, and bitterness of PP and PP with different *DH* samples were evaluated by a slightly modified method described by Garcia-Arteaga et al. [12] In brief, 10 mL sample solution was used in this test and served at random to the panelists. The samples were tested at 25 °C, in a uniformly illuminated room, by a five-member panel selected from a pool of students and staff members of our research team. The evaluation criteria were as follows: color (light to dark), taste (rough to fine), and beany flavor (serious to slight). Those sensory properties’ intensity was estimated on a five-point scale, and bitterness (light to heavy) was also evaluated on a ten-point scale. Water was provided for rinsing between samples.

### 2.4. Functional Properties

#### 2.4.1. Solubility

The solubility of PP and PP with different *DH* samples was determined by the Lowry method [23]. The specific methods are as follows: the lyophilized samples were dissolved in distilled water; then, pH was adjusted to neutral. After that, the suspension was centrifuged at 4500× *g* for 15 min. The protein content of the supernatant was determined. Bovine serum albumin (BSA) was used as the standard. Solubility was calculated according to Equation (5).

(5) $$\mathrm{Solubility}\;(\%)=\frac{\mathrm{protein}\;\mathrm{content}\;\mathrm{in}\;\mathrm{supernatant}}{\mathrm{total}\;\mathrm{protein}\;\mathrm{content}}\times 100\%$$

#### 2.4.2. Foaming Performance

The foaming properties of PP and PP with different *DH* samples were characterized at room temperature using a method described previously with some modification [24]. A fixed volume (18 mL) of protein solution was subjected to mechanical shearing (10,000 r/min, 60 s) using a high-shear mixer to generate foam (Ultra TURRAX homogenizer, T18digital, IKA, Staufen, Germany). The foamability and foam stability of each test sample were then calculated using the following Equations (6) and (7):

(6) $$\mathrm{Foamability}\;(\%)=100\times \frac{V_{0}}{18}$$

(7) $$\mathrm{Foam}\;\mathrm{stability}\;(\%)=100\times \frac{V_{20}}{V_{0}}$$

where 18 is the volume of the test sample before shearing (18 mL), *V*_0_ refers to the volume of the foam (mL) immediately after shearing, and *V*_20_ is the volume of the foam (mL) at 20 min after shearing.

#### 2.4.3. Emulsifying Performance

The emulsifying ability index (*EAI*) and emulsifying stability index (*ESI*) of PP and PP with different *DH* samples were measured according to previous papers with some modifications [25]. Then, 16 mL of protein solution was mixed with 4 mL of soybean oil, and followed by stirring at 10,000 rpm for 1 min using the high-shear mixer mentioned above. Then, 50 μL of emulsion was dispersed into 5 mL of 0.1% SDS solution at 0.5 cm at the bottom of the container, vortexed, and mixed. The absorbance values were measured at 500 nm for 0 and 10 min. *EAI* and *ESI* were calculated using the following Equations (8) and (9):

(8) $${EAI\;(m}^{2}/g)=\frac{2\times {2.303\times A}_{0}\times DF}{C\times (1-\varphi )\times \theta \times 10,000}$$

(9) $$ESI\;(\min)=\frac{A_{0}\times \Delta t}{A_{0}-A_{10}}$$

where *A*_0_ refers to the absorbance at 500 nm of the sample solution, *DF* represents the dilution factor, *C* is the protein concentration, *φ* is the oil volume fraction, and *θ* refers to the path length. In Equation (9), Δ*t* is the time difference (10 min) and *A*_10_ represents the absorbance at 500 nm of sample solution after 10 min.

### 2.5. Statistical Analyses

The experiments were conducted in triplicate. The statistical analyses were performed by SPSS 26.0 (IBM Inc., Chicago, IL, USA). Significant differences (*p* < 0.05) between the means of parameters were analyzed with one-way ANOVA test followed by Duncan’s LSD test.

## 3. Results and Discussion

### 3.1. Structure Properties

#### 3.1.1. Degree of Enzymolysis (*DH*)

**Figure 1:**
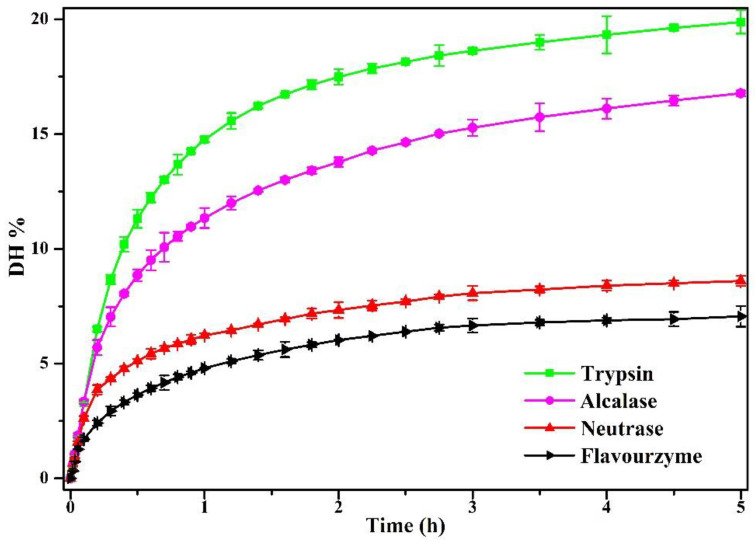
The *DH* of PP enzymolyzed by four enzymes (alcalase, neutrase, flavourzyme, and trypsin) using pH-stat method for 5 h.

In this study, pea protein (PP) was hydrolyzed by four enzymes (flavourzyme, neutrase, alcalase, and trypsin) for 5 h. As shown in Figure 1, the enzymolysis degree (*DH*) of PP increased firstly and then tended to be flat with the increase in enzymolysis time. The *DH* of PP was most intense during the first 1 h, its *DH* increased rapidly, and the *DH* of PP increased slowly after 1 h. This was consistent with Avramenko et al. [26], who pointed out that the *DH* of the lentil isolate hydrolyzed by trypsin was most rapid in the first 40 min, and then the *DH* increased slowly. Figure 1 indicated that the *DH* of PP treated by four enzymes reached 19.88% (trypsin), 16.78% (alcalase), 8.97% (neutrase), and 7% (flavourzyme) at 5 h. In the same enzymatic hydrolysis time, the enzymatic hydrolysis degree after four kinds of enzyme treatment from large to small is trypsin, alcalase, neutrase, and flavourzyme. This was closely related to the activity unit and specific restriction site of the enzyme. A similar result had been reported for pea protein isolate treated with 11 proteolytic enzymes at different enzymatic hydrolysis times [12]. Compared to previous studies, it was found that excessive enzymatic hydrolysis (*DH* ≥ 10%) showed adverse effects on the properties of PP. In addition, it was worth noting that the enzymatic hydrolysis limit of flavourzyme was 7%. Therefore, in this study, the enzymatic hydrolysates with the *DH* of 2%, 4%, 6%, and 8% (flavourzyme is 7%) of the four enzymes were selected for subsequent studies in order to clarify the effects of different enzymes on the physical and chemical properties of PP at the same *DH*.

#### 3.1.2. Intrinsic Fluorescence Spectroscopy

**Figure 2:**
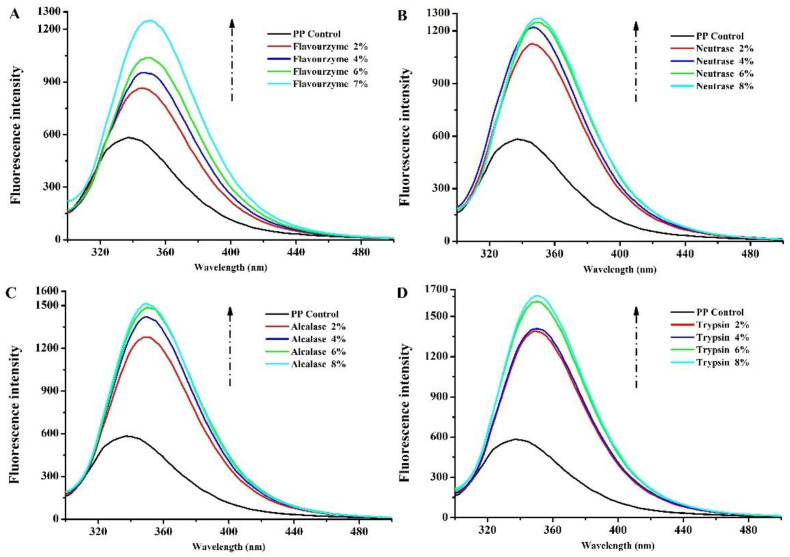
Intrinsic fluorescence of PP and PP with different *DH* samples ((**A**–**D**) are flavourzyme, neutrase, alcalase and trypsin treatment, respectively).

The intrinsic fluorescence of aromatic amino acid residues (Trp, Tyr, and Phe) is very sensitive to the microenvironment, and the emission fluorescence spectra of proteins are used to investigate the changes in their tertiary structure [27]. Therefore, the maximum fluorescence intensity and the wavelength (λmax) at the maximum fluorescence intensity are effective indicators to observe the structure and conformational changes in proteins [28]. Figure 2A–D show the fluorescence intensity of PP treated with flavourzyme, neutrase, alcalase, and trypsin, respectively. The intensities of the emission fluorescence of PP exhibited a maximum absorption at 337 nm. The λmax was affected by enzymatic treatment, and a shift in λmax to longer wavelengths (bathochromic shift) was observed for PP treated by flavourzyme enzymolysis (345.8, 345.4, 350.4, and 350.4 nm for 2%, 4%, 6%, and 7%, respectively), neutrase enzymolysis (346.8, 346.4, 350.2, and 350.4 nm for 2%, 4%, 6%, and 8%, respectively), alcalase enzymolysis (349, 349.6, 350.2, and 349.2 nm for 2%, 4%, 6%, and 8%, respectively), and trypsin enzymolysis (349.2, 350.4, 350.6, and 350.4 nm for 2%, 4%, 6%, and 8%, respectively). These results suggested that the microenvironment of chromophores in PP became more polar and hydrophilic after enzymatic treatment owing to the increased contact between the fluorophore and the aqueous medium [29]. In addition, the fluorescence intensity of PP after enzymatic hydrolysis increased significantly; the fluorescence intensity of all the four enzymes changed most significantly when *DH* increased from 0 to 2%. This indicated that the enzymatic hydrolysis process made more aromatic groups be exposed to solvent and more available to emit fluorescence [30,31]. With the increase in the *DH*, this process gradually slowed down. Among the four enzymes, trypsin-enzymolyzed PP had the highest fluorescence absorption peak. This indicated that trypsin changed the structure of PP most obviously.

#### 3.1.3. Surface Hydrophobicity

**Figure 3:**
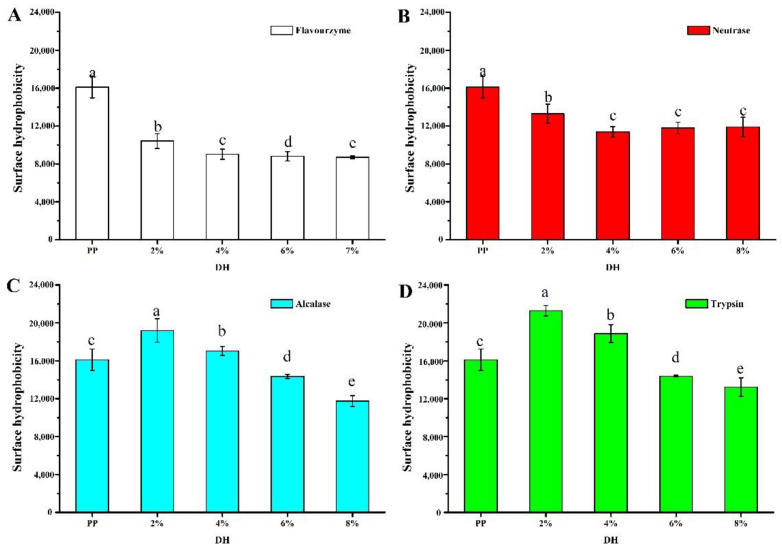
Surface hydrophobicity of PP and PP with different *DH*. The values reported represent means (*n* = 3) ± SDs and different superscript letters indicate significant difference (*p* < 0.05). ((**A**–**D**) are flavourzyme, neutrase, alcalase and trypsin treatment, respectively).

The surface hydrophobicity largely determines the protein structure and properties [32]. The changes in surface hydrophobicity of proteins after enzymatic hydrolysis were related to the type of enzyme and protein and time of enzymatic hydrolysis [33]. The surface hydrophobicity of PP after enzymatic hydrolysis by four enzymes is shown in Figure 3. Compared with PP (16,102 ± 1136), flavourzyme and neutrase showed a gradual decrease in H_0_ with increasing enzymatic hydrolysis. The H_0_ of flavourzyme 2%, 4%, 6%, and 7% was 10,418 ± 784, 9010 ± 527, 8804 ± 485, and 8693 ± 153, respectively. The H_0_ of neutrase 2%, 4%, 6%, and 8% was 19,191 ± 1234, 17,037 ± 478, 14,353 ± 222, and 11,761 ± 563, respectively. Speculatively, the above two enzymes caused the PP molecules to stretch and be partially hydrolyzed into smaller fragments, and at the same time, these small fragments would reassemble under the action of hydrophobic and disulfide bonds, resulting in a decrease in surface hydrophobic groups [34]. The H_0_ of alcalase 2%, 4%, 6%, and 8% was 19,191 ± 1234, 17,037 ± 478, 14,353 ± 222, and 11,761 ± 564, respectively. The H_0_ of trypsin 2%, 4%, 6%, and 8% was 21,274 ± 554, 18,869 ± 937, 14,387 ± 78, and 13,219 ± 987, respectively. The surface hydrophobicity of the PP treated by alcalase and trypsin tended to increase and then decrease with the increase in the *DH*, and the surface hydrophobicity of PP was greatest at a *DH* of 2%. A similar result has been reported for rice glutelin treated with trypsin [18]. The increase in hydrophobicity was due to the partial enzymatic hydrolysis that fully stretched the protein structure, thus exposing the hydrophobic sites wrapped inside the protein molecules [35]. With further increases in enzymatic hydrolysis, the hydrophobicity of the protein decreased. Enzymatic hydrolysis broke down hydrophobic regions or led to protein–protein aggregation, thereby reducing the number or surface area of hydrophobic groups exposed to water [26,34]. In general, trypsin enzymatic hydrolysis has the greatest effect on the surface hydrophobicity of PP.

#### 3.1.4. Circular Dichroism (CD) Spectroscopy Studies

As shown in Table 1, the second structure compositions of PP were 15.0% α-helix, 28.8% β-sheet, 22.1% β-turn, and 34.2% random coils. After enzymatic hydrolysis with four enzymes, the β-sheet content of enzymatic hydrolysis samples decreased with the increase in *DH*, the α-helix content increased significantly, and there was no significant change in β-turn angle and random coil. When the *DH* was 8%, the β-sheet content of PP treated by flavourzyme, neutrase, alcalase, and trypsin decreased from 28.8% to 21.7%, 20.7%, 18.5%, and 14.1%, respectively. Correspondingly, the α-helix content increased from 15.0% to 22.2%, 24.6%, 26.9%, and 32.1%, respectively. These results indicated that enzymatic hydrolysis caused perturbations of the PP secondary structure and, thus, may have an impact on the function properties of PP. Among them, the structure of β-sheet was more compact and the structure of α-helix was looser, so it can be deduced that the ordered β-sheet in the enzymatic hydrolysis products was disrupted to a more flexible and spreading α-helix structure after enzyme treatment [36]. According to the results, under the same *DH* (2%, 4%, 6%, and 8%), trypsin showed the most obvious effect on the secondary structure of PP, followed by alcalase, neutrase, and flavourzyme. The changes in the secondary structure of PP can correspond to the results of the intrinsic fluorescence and surface hydrophobicity.

#### 3.1.5. Morphology Observation

**Figure 4:**
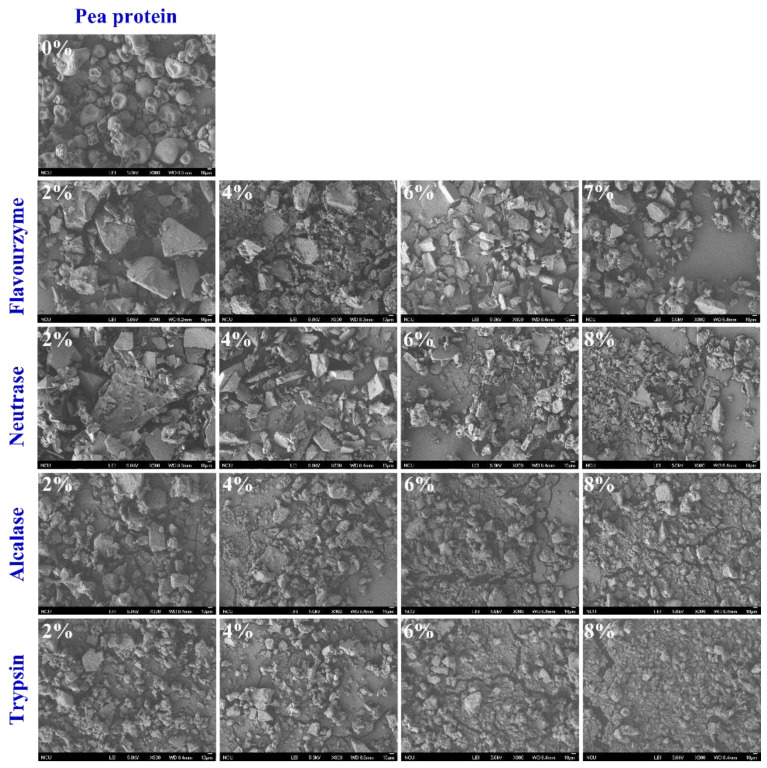
Morphology of PP and PP with different *DH* samples.

To gain insight into the effect of enzymatic hydrolysis on the change in the microstructure of PP, SEM micrographs were imaged for PP and PP with different *DH* samples. As shown in Figure 4, the natural PP is irregularly spherical with a smooth surface and a tight structure. After being treated with flavourzyme, neutrase, alcalase, and trypsin, the spherical structure of PP was broken into fragments. In addition, the microstructure of PP showed that the particles gradually became smaller with the increase in *DH*. The smaller particle size of PP made it have more chance to come into contact with water molecules during the dispersion process, thus changing the physicochemical properties of PP [29]. Furthermore, at the same *DH*, the protein particles treated by the four enzymes from small to large were trypsin, alcalase, neutrase, and flavourzyme, respectively. This result can correspond to the above result. The disruption of the protein microstructure may affect the amino acid groups embedded in the molecule and change the surface hydrophobicity, interaction forces, and secondary structure [37].

#### 3.1.6. SDS-PAGE

**Figure 5:**
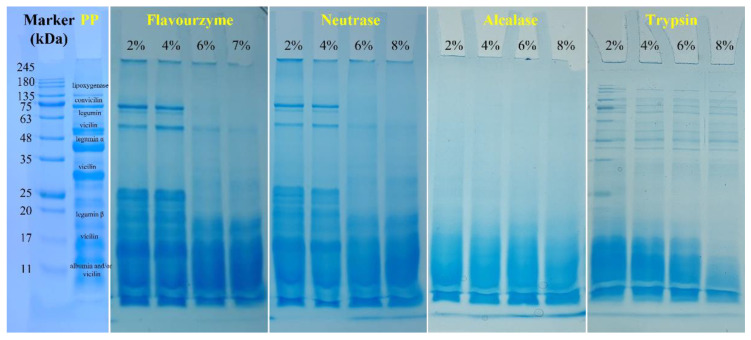
SDS-PAGE profiles of PP and PP with different *DH* samples under nonreducing conditions.

The molecular weight distribution was analyzed to investigate the effect of enzymatic hydrolysis on the PP. Figure 5 shows the SDS-PAGE electrophoresis of PP and PP with different *DH* samples. The electrophoresis pattern of PP showed multiple cleaned bands. The band at 17 kDa, 20–22 kDa, ~40 kDa, ~18 and ~50 kDa, ~60 kDa, ~75 kDa, and ~100 kDa could be attributed to 2S albumin polypeptides and/or γ-vicilin, legumin β, legumin α, vicilin, legumin, convicilin, and lipoxygenase, respectively [5]. After being treated with flavourzyme, neutrase, alcalase, and trypsin, the protein bands became progressively smaller as *DH* increased. Additionally, after enzymatic hydrolysis by flavourzyme and neutrase, the bands were clear at low *DH* (2% and 4%), but with the increase in *DH*, the protein bands were aggregated downward and the bands were gradually indistinguishable. After trypsin and alcalase treatment, the bands gradually moved toward the small molecules with the increase in *DH*, and the color of bands gradually became lighter, indicating that they kept aggregating toward the small molecule fragments after enzymatic hydrolysis. This result was consistent with the results of microstructure. It can also be shown that with the increase in *DH*, the molecular weight of PP gradually becomes smaller, which may affect the functional properties of PP. In addition, the effect of flavourzyme and neutrase on molecular weight of PP was lower than trypsin and alcalase under the same *DH*.

#### 3.1.7. Determination of Total Sulfhydryl Groups

**Figure 6:**
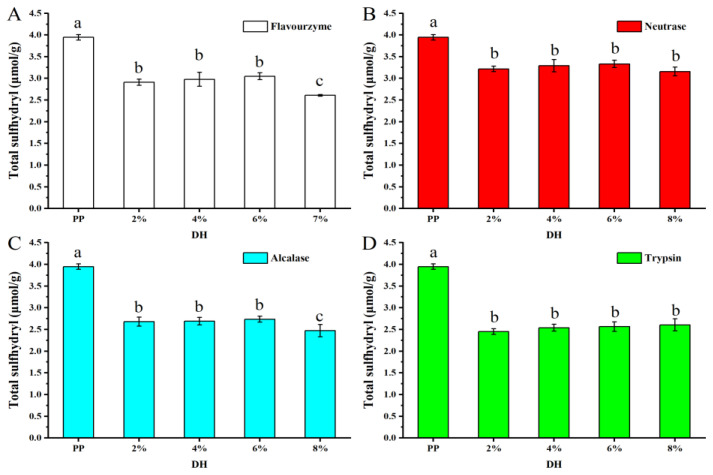
Total sulfhydryl content of PP and PP with different *DH* samples. The values reported represent means (*n* = 3) ± standard deviations and different superscript letters indicate significant difference (*p* < 0.05). ((**A**–**D**) are flavourzyme, neutrase, alcalase and trypsin treatment, respectively).

The content of sulfhydryl groups (SH) and disulfide bonds in proteins determines their rigid structure and has a significant impact on their functional properties. Figure 6 shows the variation in the total SH of samples. Compared to PP (3.94 μmol/g), the enzymatic hydrolysis samples resulted in a lower total SH. The decrease in SH content may be due to the destruction of SH within the protein molecule by enzymatic hydrolysis. As known, the spatial structure will unfold after enzymolysis; the more SH is exposed, the SH will be destroyed during enzymatic hydrolysis. As reported by Lepedda et al. [38], the SH in protein can be oxidized by enzymes, resulting the decrease in total SH. When the *DH* was 2% to 6%, the reduced SH content was the same, but when the *DH* was increased to 8%, the total SH of the samples treated by flavourzyme and alcalase further decreased significantly, which may be due to the enhanced oxidation of the SH during the enzymatic hydrolysis process. Moreover, the total SH content was reduced by trypsin > alcalase > neutrase > flavourzyme after treatment with the four enzymes, indicating that the effect of the four enzymes on the SH content and structure of PP was also trypsin > alcalase > neutrase > flavourzyme.

#### 3.1.8. Amino Acid Composition and Average Hydrophobicity Analysis

PP was considered a high-quality protein because its balanced amino acid ratio can fulfil FAO/WHO recommendations [39]. Table 2 shows the results of the amino acid composition of PP with different *DH* samples. Due to the difference in the specific cutting sites of different proteases, different amino acid compositions of peptides can be produced by enzymatic hydrolysis. As summarized in Table 2, 16 amino acids were identified and quantified among all the hydrolysates, and GLU had the highest content (it ranged from 15.70 to 18.99 g/100 g), which was similar to the amino acid composition of PP [3]. It was worth noting that the hydrophobic amino acid (HAA) (including ALA, VAL, MET, ILE, LEU, TYR, PHE, and PRO) content of PP treated by flavourzyme, neutrase, alcalase, and trypsin increased with increasing *DH* (from 2% to 8%). Meanwhile, proteolytic enzyme treatment of food protein can improve the functional and nutritional properties, but also introduce undesirable attributes [40]. Among these, bitterness is considered to be one of the main disadvantages in utilizing protein hydrolysates in food applications. In addition, sensory evaluation showed the color gradually deepened, and the roughness of the taste and the beany flavor gradually decreased with the increase in *DH* after enzymatic hydrolysis with the four enzymes. Meanwhile, the bitterness assessed by tasting was also enhanced with the increase in *DH* (data are displayed in Supplementary Materials Figure S1), and at the same *DH*, the bitterness intensity of PP treated with different enzymes was in the following order: trypsin > alcalase > neutrase > flavourzyme.

It is well known that bitter peptides are spontaneously produced during the proteolytic process and more obvious at high *DH* [41]. The degree of hydrophobicity is considered the most important predictor of peptide bitterness. So far, the Q rule proposed by Ney is still used for the relationship between hydrophobicity and bitterness of polypeptide chains [42]. As expected, the Q values tended to increase with the increase in enzymatic hydrolysis by the four enzymes, which meant that the bitterness gradually increased with the enzymatic hydrolysis. This can be explained by the results of HAA content as mentioned above. However, the Q rule found that the bitter taste was presented when the Q value was greater than 1400 cal/mol, and vice versa [41]. In this study, the Q value of all samples was below the threshold, which was not consistent with the actual bitterness of the products through tasting. Therefore, the hydrophobicity data calculated based on amino acid composition from this study do not support Ney’s Q rule as a predictor of bitterness of PP hydrolysates.

In general, the secondary structure of PP changed from ordered β-sheet to α-helix and the molecular weight of PP gradually decreased with the increase in *DH* after treatment with the four enzymes. Furthermore, the total sulfhydryl content and surface hydrophobicity of PP were changed by enzymatic hydrolysis. In addition, the bitterness of PP after enzymatic hydrolysis increased with the increase in *DH*.

### 3.2. Functional Properties

#### 3.2.1. Solubility

**Figure 7:**
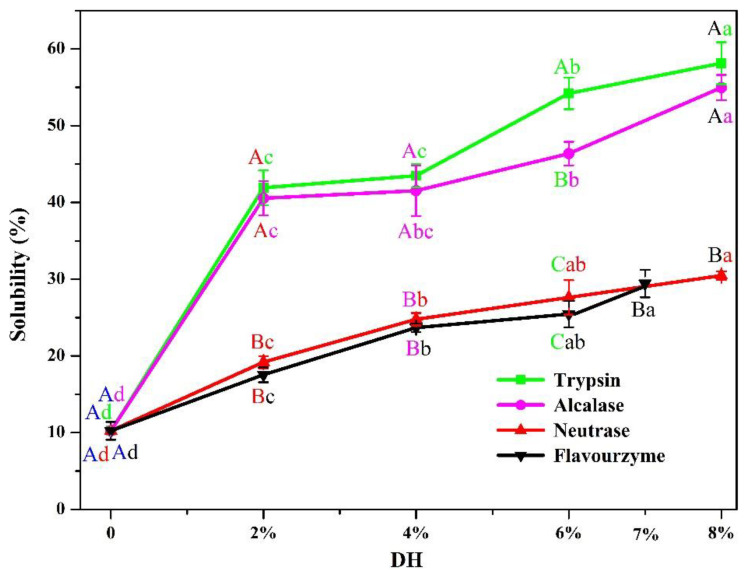
Solubility of PP and PP with different *DH* samples. Different lowercase letters are used to indicate the significant differences (*p* < 0.05) of the same enzyme under different *DH*, while different uppercase letters are used to indicate the significant differences (*p* < 0.05) of different enzymes under the same *DH*.

Solubility plays a crucial role in the functionality of the protein and protein-based systems including gels, emulsions, and foams [43]. Especially for plant protein, boosted properties can benefit from the increase in solubility [44]. The solubility of PP and PP with different *DH* samples is shown in Figure 7. Compared with PP (10.23%), the solubility of PP gradually increased with the increase in *DH*. The solubility of flavourzyme 2%, 4%, 6%, and 7% was 17.54%, 23.68%, 25.46%, and 29.44%, respectively. The solubility of neutrase 2%, 4%, 6%, and 8% was 19.18%, 24.78%, 27.62%, and 30.47%, respectively. The solubility of alcalase 2%, 4%, 6%, and 8% was 40.55%, 41.52%, 46.37%, and 54.97%, respectively. The solubility of trypsin 2%, 4%, 6%, and 8% was 41.91%, 43.49%, 54.22%, and 58.14%, respectively. The increased solubility may be due to the decreased molecular size of PP and smaller peptides when the PP was hydrolyzed. This was consistent with the results of SDS-PAGE. In addition, the effect of different enzymes on PP at the same *DH* was different. From the experimental results, the degree of change induced by the four enzymes on PP solubility was: trypsin > alcalase > neutrase > flavourzyme. This meant that trypsin had the best enzymatic hydrolysis effect on PP of all the enzymes.

#### 3.2.2. Foaming Performance

**Figure 8:**
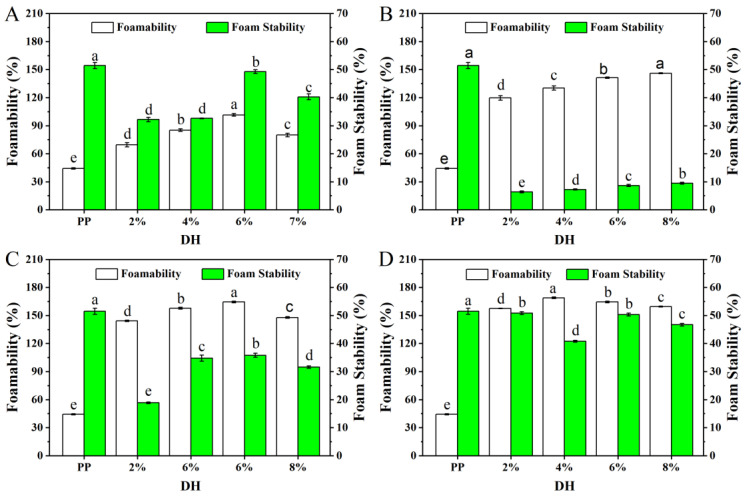
The foamability and foam stability of PP and PP with different *DH* samples ((**A**–**D**) are flavourzyme, neutrase, alcalase, and trypsin treatment, respectively). The values reported represent means (*n* = 3) ± SDs and different superscript letters indicate significant difference (*p* < 0.05).

The foaming capacity and foaming stability of PP and PP with different *DH* samples are shown in Figure 8. The foaming ability of the enzymatically digested PP was significantly higher than the native PP, which was consistent with the solubility results. Solubility is a prerequisite for protein-foaming ability, and the protein should first dissolve in the aqueous phase and then rapidly stretch to form a dense layer of protein molecules around the air and foaming [45]. The foaming ability and foaming stability of protein was also related to the *DH*. After the four enzymes’ treatment, the foaming ability of PP treated with flavourzyme, alcalase, and trypsin showed an increasing trend and then decreased, reaching the best levels at 6% (101.48%), 6% (164.44%), and 4% (168.88%) of *DH*, respectively. The foaming ability of PP treated by neutrase increased gradually. Speculatively, limited enzymatic hydrolysis produced peptides with balanced hydrophilic/hydrophobic groups, and the amphiphilic parts of proteins and peptides have a stronger capacity to decrease the surface tension at the air–water interface. However, high *DH* usually had an adverse effect on foaming properties. On the one hand, the increase in *DH* increased the protein solubility, but the best structure of protein spheres was destroyed, thus resulting in a reduction in the foaming ability. On the other hand, excessive enzymolysis made the hydrolysates more hydrophilic, disturbing the hydrophilic/hydrophobic balance, which led to the decreased foam formation ability [46]. In addition, after the four enzymes (flavourzyme, neutrase, alcalase, and trypsin) treatment, the foaming stability of PP was decreased compared with native PP.

#### 3.2.3. Emulsifying Performance

**Figure 9:**
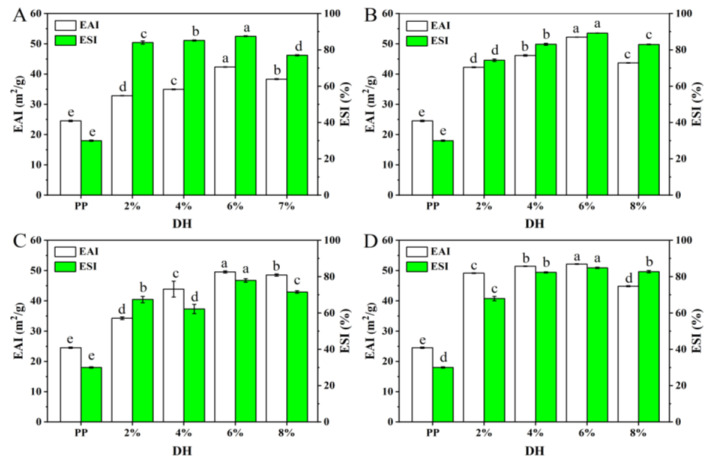
The *EAI* and *ESI* of PP and PP with different *DH* samples ((**A**–**D**) are flavourzyme, neutrase, alcalase, and trypsin treatment, respectively). The different superscript letters of same index indicate significant difference (*p* < 0.05).

Proteins are a kind of natural emulsifier that are widely used in food emulsion preparation due to their amphiphilic nature. The emulsifying property of protein represents the ability of protein molecules in an emulsion to adsorb to the oil–water interface and is usually characterized by emulsifying activity and emulsifying stability [47]. Usually, the stronger the emulsification, the more stable the emulsion which forms and the less likely it is to form an aggregation. Figure 9 shows the changes in the emulsifying properties and emulsion stability of PP and PP with different *DH* samples. As shown in Figure 9, the emulsification ability and emulsion stability of PP treated with the four enzymes were significantly improved, which may be related to the increased solubility and peptide chain flexibility of the PP enzymatic hydrolysis products. All four enzymes showed an increasing trend and then a decrease in emulsifying properties as the *DH* increased, and all of them were highest at a *DH* of 6%. When the *DH* was 6%, the *EAI* of PP treated by flavourzyme, neutrase, alcalase, and trypsin increased from 24.55 to 42.36, 52.22, 49.55, and 52.16 m^2^/g, respectively, while the *ESI* increased from 30.02 to 87.54, 89.23, 77.93, and 84.88%, respectively. This may be due to the fact that excessive enzymolysis further reduces the molecular weight of the protein and reduces the amphiphilicity of the peptide, thus inhibiting the interaction between protein molecules at the oil–water interface and reducing the viscoelasticity of the interface membrane [47,48]. In addition, it may be due to the reduced charge repulsion between low-molecular-weight peptides preventing the proteins from either stretching or rearranging at the interface [49].

Based on the above results and analysis, the solubility of PP gradually increased with the increase in *DH* after treatment with the four enzymes. Furthermore, the foaming capacity of PP treated with four enzymes was significantly improved, and the best foaming capacity was observed for flavourzyme, alcalase, trypsin, and neutrase with *DH*s of 6%, 6%, 4%, and 8%, respectively. In addition, the emulsification ability and emulsion stability of PP treated with the four enzymes were significantly improved, and all of them were the highest at a *DH* of 6%. Overall, the functional properties of PP treated with the four enzymes were the best when the *DH* was 6%.

## 4. Conclusions

The present study aimed to investigate the effects of four different enzymes on the structure and functional properties of PP at the same *DH*. At the same *DH*, trypsin had the strongest effect on improving the properties of pea protein, followed by alcalase, neutrase, and flavourzyme. Trypsin-treated PP had smaller surrounding particles, smaller molecular weight, less total sulfhydryl content, higher solubility, and higher fluorescence intensity. With the increase in *DH*, the improvement effect on the foaming and emulsification of PP tended to increase and then decrease and reached the best value at 6% *DH*. This study provided important information about moderate enzymatic hydrolysis, which can broaden the application of pea protein in plant-based proteins.

## Figures and Tables

**Table 1:** Secondary structure content of PP and PP with different *DH* samples determined by circular dichroism.

**Protein**	**Flavourzyme**				**Protein**	**Neutrase**			
	α-Helix (%)	β-Sheet (%)	β-Turn (%)	Random (%)		α-Helix (%)	β-Sheet (%)	β-Turn (%)	Random (%)
PP	15.0	28.8	22.1	34.2	PP	15.0	28.8	22.1	34.2
2%	20.1	24.4	19.6	35.9	2%	21.2	23.5	20.4	34.9
4%	20.5	23.8	19.8	35.9	4%	22.5	22.8	19.0	35.7
6%	21.6	22.4	19.8	36.2	6%	23.0	22.2	18.2	36.6
7%	22.2	21.7	19.7	36.4	8%	21.2	23.5	20.4	34.9
**Protein**	**Alcalase**				**Protein**	**Trypsin**			
	α-Helix (%)	β-Sheet (%)	β-Turn (%)	Random (%)		α-Helix (%)	β-Sheet (%)	β-Turn (%)	Random (%)
PP	15.0	28.8	22.1	34.2	PP	15.0	28.8	22.1	34.2
2%	24.5	21.7	19.5	34.3	2%	25.9	20.8	19.1	34.2
4%	24.7	21.4	19.5	34.4	4%	27.9	19.3	19.0	33.8
6%	25.6	20.3	19.0	35.1	6%	29.2	16.9	19.9	34.0
8%	26.9	18.5	18.3	36.3	8%	32.1	14.1	19.0	34.8

**Table 2:** Amino acid composition of PP with different *DH* samples treated with flavourzyme, neutrase, alcalase, and trypsin (g/100 g). HAA: hydrophobic amino acids.

Amino Acid	Flavourzyme				Neutrase				Alcalase				Trypsin			
	2%	4%	6%	7%	2%	4%	6%	8%	2%	4%	6%	8%	2%	4%	6%	8%
**ASP**	7.79 ± 0.01 a	8.66 ± 0.02 c	8.09 ± 0.00 b	8.61 ± 0.02 c	9.09 ± 0.02 a	9.17 ± 0.01 b	9.58 ± 0.03 c	9.70 ± 0.01 d	10.24 ± 0.03 c	10.02 ± 0.01 a	10.13 ± 0.02 b	10.18 ± 0.03 b	9.58 ± 0.01 a	9.70 ± 0.02 b	9.58 ± 0.04 a	9.73 ± 0.02 b
**THR**	2.05 ± 0.01 a	2.25 ± 0.00 d	2.18 ± 0.01 c	2.07 ± 0.02 b	2.76 ± 0.02 a	2.89 ± 0.00 b	3.06 ± 0.01 c	3.10 ± 0.01 d	3.05 ± 0.01 a	3.05 ± 0.03 a	3.04 ± 0.01 a	3.24 ± 0.02 b	3.11 ± 0.00 b	3.06 ± 0.02 a	3.13 ± 0.01 b	3.18 ± 0.03 c
**SER**	3.36 ± 0.00 c	3.38 ± 0.01 c	2.98 ± 0.02 b	2.67 ± 0.00 a	3.85 ± 0.01 a	3.83 ± 0.02 a	3.93 ± 0.01 b	4.04 ± 0.02 c	4.01 ± 0.01 c	3.86 ± 0.02 a	3.82 ± 0.01 a	3.95 ± 0.02 b	4.05 ± 0.03 c	3.92 ± 0.01 a	3.93 ± 0.01 a	3.98 ± 0.01 b
**GLU**	18.59 ± 0.05 c	18.99 ± 0.03 d	18.05 ± 0.06 b	17.39 ± 0.03 a	16.73 ± 0.03 d	15.89 ± 0.02 a	16.25 ± 0.02 b	16.62 ± 0.01 c	18.52 ± 0.02 d	17.52 ± 0.03 c	16.49 ± 0.01 a	16.83 ± 0.04 b	16.99 ± 0.01 d	15.90 ± 0.02 c	15.70 ± 0.01 a	15.78 ± 0.02 b
**PRO**	2.68 ± 0.02 a	3.00 ± 0.01 b	3.13 ± 0.01 c	3.18 ± 0.01 d	3.19 ± 0.02 a	3.25 ± 0.01 b	3.36 ± 0.01 c	3.40 ± 0.00 d	3.44 ± 0.01 a	3.45 ± 0.02 a	3.50 ± 0.00 b	3.63 ± 0.01 c	3.42 ± 0.02 a	3.42 ± 0.01 a	3.43 ± 0.00 a	3.44 ± 0.01 a
**GLY**	2.75 ± 0.01 a	2.90 ± 0.00 b	2.89 ± 0.02 b	2.92 ± 0.01 b	2.95 ± 0.02 a	2.99 ± 0.00 b	3.08 ± 0.01 c	3.12 ± 0.01 d	3.22 ± 0.00 c	3.17 ± 0.02 b	3.09 ± 0.01 a	3.31 ± 0.01 d	3.15 ± 0.00 b	3.09 ± 0.01 a	3.13 ± 0.02 b	3.16 ± 0.01 b
**ALA**	2.55 ± 0.00 a	2.82 ± 0.02 b	2.89 ± 0.00 c	2.98 ± 0.01 d	3.08 ± 0.01 a	3.15 ± 0.01 b	3.29 ± 0.01 c	3.31 ± 0.01 c	3.30 ± 0.01 a	3.33 ± 0.01 a	3.39 ± 0.01 b	3.45 ± 0.01 c	3.28 ± 0.01 a	3.35 ± 0.01 b	3.36 ± 0.01 b	3.39 ± 0.02 b
**VAL**	2.94 ± 0.01 a	3.24 ± 0.01 b	3.33 ± 0.03 c	3.54 ± 0.02 d	3.45 ± 0.01 a	3.56 ± 0.01 b	3.67 ± 0.02 c	3.81 ± 0.02 d	3.68 ± 0.02 a	3.71 ± 0.01 a	3.72 ± 0.01 a	3.84 ± 0.02 b	3.64 ± 0.01 a	3.70 ± 0.02 b	3.74 ± 0.00 c	3.77 ± 0.01 d
**MET**	0.05 ± 0.00 a	0.19 ± 0.01 b	0.22 ± 0.01 b	0.25 ± 0.00 c	0.33 ± 0.01 a	0.40 ± 0.01 b	0.42 ± 0.00 b	0.45 ± 0.01 c	0.34 ± 0.01 a	0.36 ± 0.00 a	0.43 ± 0.00 b	0.49 ± 0.01 c	0.42 ± 0.01 a	0.45 ± 0.01 a	0.46 ± 0.01 a	0.54 ± 0.00 b
**ILE**	2.76 ± 0.02 a	3.03 ± 0.03 b	3.16 ± 0.01 c	3.33 ± 0.01 d	3.19 ± 0.01 a	3.32 ± 0.02 b	3.42 ± 0.01 c	3.46 ± 0.02 c	3.40 ± 0.01 a	3.45 ± 0.02 b	3.46 ± 0.01 b	3.61 ± 0.02 c	3.31 ± 0.02 a	3.33 ± 0.01 a	3.36 ± 0.01 b	3.40 ± 0.01 c
**LEU**	4.72 ± 0.01 a	5.43 ± 0.01 b	5.63 ± 0.03 c	5.81 ± 0.01 d	5.75 ± 0.02 a	5.89 ± 0.01 b	6.09 ± 0.02 c	6.17 ± 0.01 d	6.15 ± 0.01 a	6.19 ± 0.02 a	6.25 ± 0.02 b	6.34 ± 0.02 c	6.14 ± 0.01 a	6.15 ± 0.03 a	6.31 ± 0.02 b	6.44 ± 0.01 c
**TYR**	1.32 ± 0.01 a	1.56 ± 0.00 b	1.69 ± 0.01 c	1.87 ± 0.00 d	1.83 ± 0.00 a	2.03 ± 0.01 b	2.17 ± 0.01 c	2.19 ± 0.01 c	2.17 ± 0.00 a	2.34 ± 0.01 b	2.36 ± 0.00 b	2.54 ± 0.01 c	2.37 ± 0.01 a	2.41 ± 0.01 b	2.57 ± 0.01 c	2.61 ± 0.02 d
**PHE**	2.91 ± 0.01 a	3.80 ± 0.02 b	3.90 ± 0.00 c	4.03 ± 0.03 d	3.92 ± 0.01 a	4.03 ± 0.01 b	4.18 ± 0.01 c	4.20 ± 0.01 c	4.08 ± 0.01 a	4.15 ± 0.01 b	4.16 ± 0.01 b	4.30 ± 0.01 c	4.10 ± 0.00 a	4.13 ± 0.01 b	4.20 ± 0.01 c	4.25 ± 0.01 d
**LYS**	5.61 ± 0.03 b	5.79 ± 0.01 c	5.63 ± 0.03 b	5.06 ± 0.02 a	5.99 ± 0.02 a	5.95 ± 0.01 a	6.21 ± 0.02 b	6.21 ± 0.01 b	6.42 ± 0.02 c	6.29 ± 0.01 b	5.99 ± 0.02 a	6.26 ± 0.03 b	6.08 ± 0.02 c	5.83 ± 0.03 a	5.83 ± 0.01 a	5.94 ± 0.00 b
**HIS**	1.01 ± 0.00 b	1.16 ± 0.01 c	0.92 ± 0.00 a	0.90 ± 0.02 a	1.13 ± 0.00 b	1.12 ± 0.01 b	1.13 ± 0.00 b	1.07 ± 0.00 a	1.07 ± 0.00 b	1.09 ± 0.01 b	1.04 ± 0.01 a	1.12 ± 0.01 b	1.07 ± 0.00 a	1.09 ± 0.01 b	1.06 ± 0.00 a	1.10 ± 0.01 b
**ARG**	6.13 ± 0.03 c	6.45 ± 0.03 d	5.44 ± 0.01 b	4.98 ± 0.02 a	7.26 ± 0.02 b	7.16 ± 0.02 a	7.39 ± 0.01 c	7.49 ± 0.01 d	7.65 ± 0.02 c	7.42 ± 0.03 b	7.09 ± 0.01 a	6.41 ± 0.02 b	7.47 ± 0.01 c	7.11 ± 0.00 b	7.07 ± 0.01 a	7.11 ± 0.03 ab
**HAA**	19.92 ± 0.03 a	23.08 ± 0.02 b	23.94 ± 0.06 c	24.99 ± 0.02 d	24.73 ± 0.05 a	25.62 ± 0.03 b	26.59 ± 0.01 c	26.99 ± 0.02 d	26.58 ± 0.05 a	26.99 ± 0.05 b	27.61 ± 0.01 c	28.18 ± 0.03 d	26.50 ± 0.03 a	27.13 ± 0.02 b	27.43 ± 0.03 c	27.83 ± 0.05 d
**Q value**	1039.94 ± 1.21 a	1074.08 ± 0.86 b	1108.16 ± 1.11 c	1126.82 ± 2.01 d	1089.13 ± 1.01 a	1101.133 ± 2.11 b	1104.35 ± 1.33 b	1108.58 ± 0.56 c	1083.81 ± 1.51 a	1098.21 ± 2.11 b	1104.91 ± 0.96 c	1111.09 ± 1.71 d	1094.78 ± 1.44 a	1105.858 ± 1.66 b	1110.83 ± 1.13 c	1112.40 ± 0.56 c

The same enzyme and different letters on the same row indicate significant differences (*p* < 0.05).

## Data Availability

Data are contained within the article.

## Supplementary Materials

The following supporting information can be downloaded at: https://www.mdpi.com/article/10.3390/foods11152368/s1, Figure S1: Sensory evaluation results of PP and PP with different *DH* samples.
