# Supplementary material for: Effects of Moderate Enzymatic Hydrolysis on Structure and Functional Properties of Pea Protein
Source: Foods. 2022 Aug 7;11(15):2368. doi: 10.3390/foods11152368 (PMC9368430; doi:10.3390/foods11152368)
Supplement: Supplementary file 1 [file foods-11-02368-s001.zip › foods-1819866-supplementary.pdf]

Supplementary materials Figure S1

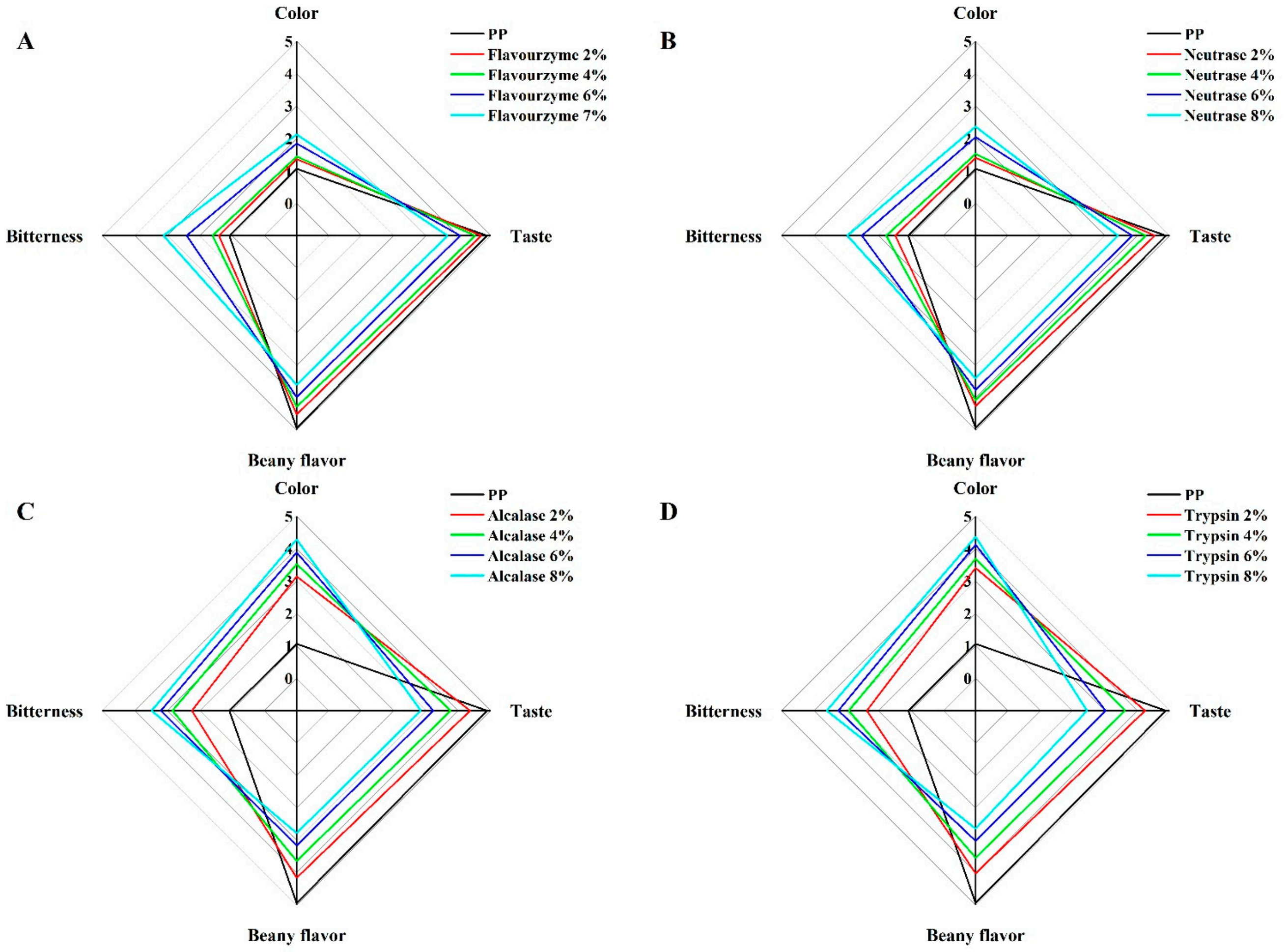

Figure S1 Sensory evaluation results of PP and PP with different DH samples. A: Flavourzyme, B: Neutrase, C: Alcalase, D: Trypsin.
